# Salmagundi

**Published:** 1885-05

**Authors:** 


### SALMAGUNDI.

EDITED BY E. G. BETTY, D. D. S.

Soothing Syrups and Popular Remedies.—Opium forms the basis of innumerable remedies, and very effective remedies, sold under titles altogether reassuring and misleading. Nearly all soothing syrups and powders, for example—" mothers' blessings" and infants' curses—are really opiates. These are known or suspected by most well-informed people. What is less generally known is that nine in ten of the popular remedies for catarrh, bronchitis, cough, cold, and asthma are also opiates. So powerful, indeed, is the effect of opium upon the lining membrane of the lungs and air passages, so difficult is it to find an effective substitute, that the efficacy, at least the certain and rapid efficacy, of any specific remedy for cold whose exact nature is not known affords strong grounds for suspecting the presence of opium. Many chemists are culpably, almost criminally, reckless, and not a few culpably ignorant in this matter. An experienced man bought from a fashionable West End shop a box of cough lozenges, pleasant to the taste and relieving a severe cough with a wonderful rapidity. Familiar with the influence of opium on the stomach and spirits, he was sure before he had sucked half a dozen of the lozenges that he had taken a dose powerful enough to affect his accustomed system, and strong enough to poison a child and do serious harm to a sensitive adult. Yet the lozenges were sold without warning or indication of their character; few people would have taken a special precaution to keep them out of the way of children, and the box, falling into the hands of a heedless or disobedient child, might have poisoned a whole nursery.—*National Review.*

Dentistry in Africa.—The negroes of the lower Congo region, although not very cleanly, generally take good care of their teeth, washing them with water after each meal, using their fingers in lieu of the brush.

The different tribes sharpen their teeth with stones, using them as files and so cut them into various shapes other than the natural. One tribe grind the incisors wedge-shaped, another trapezoid, while another makes them resemble the teeth of a saw. It may be interesting and satisfactory for some to know that these tooth-decorating tribes are strictly vegetarians.—*Translation.*

---

THERE are no such things as white teeth, as can be proved by contrasting those called white with snow, raw cotton, or marble. Roughly, teeth can be divided, as regards color, into blue, gray, and yellow, with hundreds of different shades. The apparent whiteness is due to the complexion and hue of the lips; and the "glistening ivories" of the negro are, as a rule, so exceedingly yellow that they would disfigure a white person. It is the black skin, hair, and eyes that make them look white.—*Clipping.*

---

"A Newfoundland dog, says a New York paper, swallowed a a set of artificial teeth belonging to his mistress, whose little nephew induced a sympathizing druggist to go to work on the animal with emetics, with the result of having the teeth restored to the owner, without her becoming aware of the manner of their loss."

---

"THAT is a lovely horseshoe ornament," remarked a young man, while calling on a lady of his acquaintance, pointing to a horseshoe over the door decorated with forget-me-nots and daisies. "Pooh! that's no horseshoe; that's Emma's old false teeth that she trimmed up," shouted the young brother who was behind the piano.

---

GENERAL GRANT AND HIS TOOTH BRUSH.—In the April number of the *Independent Practitioner*, Dr. Frank Abbott publishes an account of the condition in which he found the General's mouth, and the possible, if not probable, cause of the throat trouble being traceable to broken teeth and large incrustations of salivary calculus, instead of the cigar, as has been so frequently stated. Dr. Abbott removed a great deal of tartar, as well as several upper molars that were in a bad state.

It appears that General Grant has neglected his mouth of late years (which smokers generally do) as the following testimony of the Hon. E. B. Washburne shows that at one time, even in the heat of vigorous campaigns, the great soldier never forgot the care of his mouth, as at that time the habit of smoking had been acquired but about two years previous, at the battle of Shiloh.

"When he left his headquarters, at Smith's plantation, below Vicksburg, to enter on that great campaign, he did not take with him the trappings and paraphernalia so common to many military men. As all depended on quickness of movement, and as it was important to be encumbered with as little baggage as possible, he set an example to all under him. He took with him neither a horse, nor an orderly, nor a servant, a camp chest, an overcoat, nor blanket, nor even a clean shirt. *His entire baggage for six days—I was with him at that time—was a tooth brush.* He fared like the commonest soldier in his command, partaking of his rations and sleeping upon the ground, with no covering excepting the canopy of heaven. How could such a soldier fail to inspire confidence in an army, and to lead it to victory and to glory?"

---

Our esteemed friend, Dr. W. C. Barrett, of the *Independent Practitioner*, in an editorial in the April number of that excellent journal, on "Cocaine in Dental Practice," endeavors to give the *modus operandi* of the anæsthetic:

He puts it thus: "All true anæsthetics paralyze nerve tissue, commencing at the periphery and proceeding toward the nerve centers. Their specific effect is upon the nerves themselves, and they are brought in contact with them by being carried through the arterial supply, and hence their first impression is upon the terminal filaments. Local anæsthetics, on the contrary, are not distributed through the general circulation, but are placed in direct contact with the peripheral ends of the nerves at once, and manifest their peculiar properties only upon the nerves which are thus brought within their immediate influence.

Cocaine is such a local anæsthetic, and when tissues having a nervous supply are submitted to its action, either by hypodermic injection or through absorption from topical application, its regular characteristic effects are manifested."

Now, then, the point is *how* does cocaine produce anæsthesia?

The remedy undoubtedly acts upon nervous tissue, and for that reason will not become an obtunder of sensitive dentine, but is it reasonable to suppose that it paralyzes nerve tissue in any other manner than by causing anaemia? We would like to hear from our readers on that point.

---

We received a card of invitation to the wedding of Katie L., daughter of Dr. and Mrs. H. M. Reid, formerly of Cincinnati, but now of Minneapolis, Minn., and Mr. Edward B. Clement. The event was fixed for April 15th, and for aught we know to the contrary, has passed off with great pleasure to all. Not being able to attend the ceremony, we must rest content with wishing the young couple good luck and *bon voyage*.

---

The next annual meeting of the American Dental Association will be held in Minneapolis, Minnesota, first week in August, and it behooves all us Western " backwoodsmen " to be present in force and make the occasion a grand success. Put the date down in your " di-a-wy, oh--oh--ye-yes," and so don't forget it.

